# Evaluation of an angiotensin Type 1 receptor blocker on the reconsolidation of fear memory

**DOI:** 10.1038/s41398-020-01043-6

**Published:** 2020-10-27

**Authors:** Adam P. Swiercz, Laxmi Iyer, Zhe Yu, Allison Edwards, N. M. Prashant, Bryan N. Nguyen, Anelia Horvath, Paul J. Marvar

**Affiliations:** 1 Department of Pharmacology and Physiology, George Washington University, Washington, DC 20052 USA; 2 Computational Biology Institute, George Washington University, Washington, DC 20052 USA; 3 Department of Psychiatry and Behavioral Sciences, George Washington University, Washington, DC 20052 USA

**Keywords:** Long-term memory, Physiology

## Abstract

Inhibition of the angiotensin type 1 receptor (AT_1_R) has been shown to decrease fear responses in both humans and rodents. These effects are attributed to modulation of extinction learning, however the contribution of AT_1_R to alternative memory processes remains unclear. Using classic Pavlovian conditioning combined with radiotelemetry and whole-genome RNA sequencing, we evaluated the effects of the AT_1_R antagonist losartan on fear memory reconsolidation. Following the retrieval of conditioned auditory fear memory, animals were given a single intraperitoneal injection of losartan or saline. In response to the conditioned stimulus (CS), losartan-treated animals exhibited significantly less freezing at 24 h and 1 week; an effect that was dependent upon memory reactivation and independent of conditioned cardiovascular reactivity. Using an unbiased whole-genome RNA sequencing approach, transcriptomic analysis of the basolateral amygdala (BLA) identified losartan-dependent differences in gene expression during the reconsolidation phase. These findings demonstrate that post-retrieval losartan modifies behavioral and transcriptomic markers of conditioned fear memory, supporting an important regulatory role for this receptor in reconsolidation and as a potential pharmacotherapeutic target for maladaptive fear disorders such as PTSD.

## Introduction

Life-threatening traumatic events (e.g., military combat, assault, or natural disaster) can lead to the formation of maladaptive fear memories and the development of mental health disorders such as posttraumatic stress disorder (PTSD). The renin-angiotensin system (RAS) has been identified as a potential pharmacotherapeutic target for PTSD as retrospective clinical studies have shown that treatment with angiotensin-converting enzyme inhibitors (ACEi) and angiotensin receptor blockers (ARBs) are associated with fewer PTSD symptoms^1,2^. Pre-clinical research demonstrates that peripheral AT_1_R inhibition with losartan^3–5^ or deletion of AT_1_R from select neuronal populations facilitates fear memory extinction^6^. More recent evidence in humans indicates that losartan improves early threat discrimination and facilitates threat processing^7^, in addition to accelerating fear extinction^8^ and modifying aversive learning^9^. Despite these recent observations in both humans and rodents^10^, in addition to earlier literature describing a role for angiotensins in learning and memory^11,12^, many of the underlying neurobiological mechanisms remain unknown.

Memory retrieval can initiate a distinct, protein synthesis-dependent mnemonic process called reconsolidation, during which reactivated memories become temporarily labile and susceptible to updating^13^. Reconsolidation provides a time-limited window of vulnerability to selectively weaken or enhance a previously consolidated memory^14^. Because interference with reconsolidation can have amnesic effects on the reactivated memory, this process could potentially be manipulated to benefit a range of psychiatric conditions, including PTSD, obsessive compulsive disorder, delusions, and hallucinations^15^.

Interestingly, increases in brain angiotensin II (Ang II) in response to water deprivation can strengthen memory by facilitating reconsolidation in an invertebrate animal model^16^. A similar strengthening of contextual fear memory reconsolidation by Ang II-mediated AT_1_R activation in the dorsal hippocampus occurs in rats^17^. If endogenous Ang II is responsible for reconsolidation enhancement, it may be possible to weaken long-term memories by blocking AT_1_R activation during the window of reconsolidation. Therefore, using classic Pavlovian conditioning combined with telemetry and whole-genome RNA sequencing, we sought to further examine the neurobiological mechanisms of AT_1_R inhibition of fear memory. We hypothesized that post-retrieval blockade of AT_1_R would modify reconsolidation and key central transcriptomic pathways, leading to a reduction in fear responses to a previously conditioned stimulus.

Our findings indicate that losartan, administered shortly after memory retrieval, reduces freezing behavior independent of cardiovascular reactivity while altering differential gene expression patterns in the amygdala. These studies expand upon our currently limited understanding of the mechanisms linking angiotensin receptors to fear-related memory^3,4,6,18,19^.

## Materials and methods

All procedures were approved by the Institutional Animal Care and Use Committee at The George Washington University and were in compliance with National Institutes of Health guidelines. Adult male (3–4 months old) C57BL/6J mice from Jackson Laboratory (Bar Harbor, ME, United States) were used for all experiments. Animals were individually housed in temperature and humidity-controlled polyethylene cages on a 12 h light/dark cycle (lights off at 7 p.m.) and supplied with food and water ad libitum. The number of mice used in our behavioral and telemetry experiments was arrived at based on previous published data using the average number of animals needed to obtain statistical significance, obtaining 80% power to detect a difference between *p* = 0.01 to *p* = 0.05.

### Telemetry

HDX-11 transmitters (Data Sciences International (DSI); St. Paul, MN) were subcutaneously implanted as previously described^20^. A blood pressure catheter was placed into the left carotid artery and advanced to the aortic arch. Animals recovered for 14 days after surgery before beginning behavioral experiments. During telemetry recording, blood pressure signals were sampled at a rate of 500 Hz. Blood pressure, heart rate, and activity data were continuously collected during memory retrieval and memory testing. Blood pressure data were analyzed using Ponemah software version 6.3 (DSI).

### Drugs

The AT_1_R antagonist losartan (Sigma-Aldrich; St. Louis, MO) was dissolved in sterile saline for intraperitoneal injection (10 mg/kg). The total injection volume was 0.2 ml per animal. This dose was selected based on previous studies demonstrating an effect on memory extinction^4^.

### Pavlovian fear conditioning

Auditory fear conditioning was performed as previously described^20,21^. Mice were individually habituated with the experimenter and conditioning chamber for 20 and 40-min sessions on days 1 and 2. Fear conditioning consisted of five trials of a conditioned stimulus (CS) tone (30 s, 6 kHz, 75 dB) co-terminating with an unconditioned stimulus (US) foot shock (0.5 mA, 0.5 s,) spaced by 3 min 30 s inter-trial intervals. All experiments were conducted in the light phase and freezing behavior was calculated automatically using Freeze Frame 3.2 (Actimetrics; Wilmette, IL) and investigator was blinded to treatment during subsequent data analysis.

### Retrieval

**Fig. 1:**
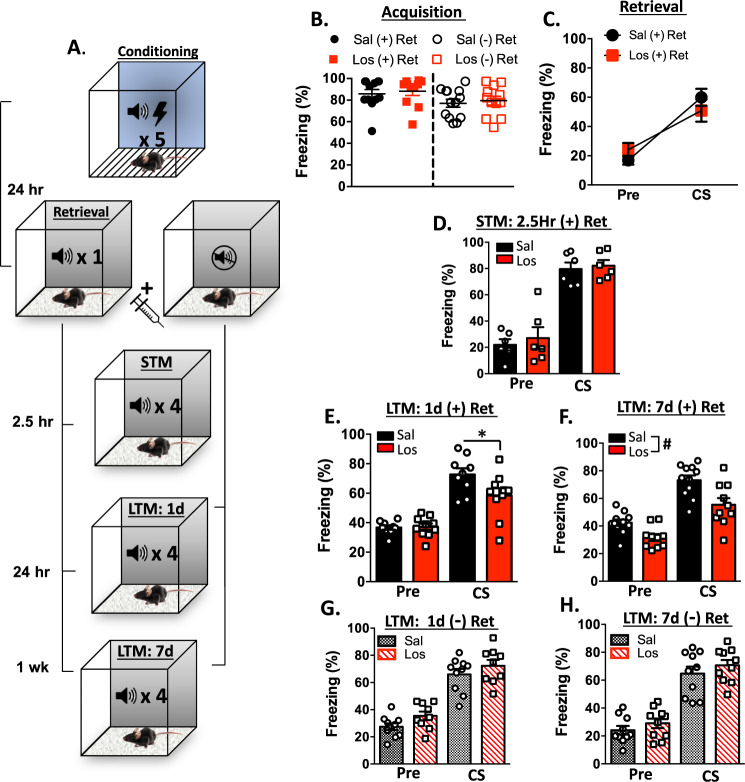
Post-retrieval losartan reduces freezing behavior. **a** Schematic of the conditioning, retrieval, and testing protocol. **b** Average freezing during the 5th CS presentation of fear conditioning. **c** Freezing behavior before and during the 1CS retrieval cue. Non-retrieval groups (−) did not receive cue exposure. Freezing response to 4 CS presentations during the (**d**) 2.5 h (STM) (**e**) 24 h (LTM-1d), and (**f**) 1 week (LTM-7d) memory tests in retrieval groups. Freezing response to 4 CS presentations during the (**g**) LTM-1d and (**h**) LTM-7d tests in non-retrieval groups. (*n* = 11 losartan *n* = 13 saline group, #saline vs. losartan *p* < 0.05, *Bonferroni post hoc *p* < 0.05).

Twenty-four hours after fear conditioning, animals were re-exposed to the CS to reactivate the memory and initiate reconsolidation^22^. The fear conditioning chamber was modified by replacing the shock grid with a clear Plexiglas floor, covering the clear walls of the chamber with patterned construction paper, and changing the scent of the chamber with peppermint oil. A baseline period of 120 s, during which the Pre-CS percent freezing average was calculated, preceded the presentation of a single 30 s CS in the retrieval (+) group, or no CS in the non-retrieval (−) group. All animals received a peripheral injection of either saline (0.2 ml ip) or losartan (10 mg/kg ip) 10 min after being removed from the retrieval chamber. The behavioral protocol is depicted in Fig. 1a. Animals failing to respond the retrieval cue (less than 10% freezing) were removed from the study, resulting in the exclusion of two mice from further analysis.

### Testing

The experiments were broken up into two randomized primary behavioral groups. In the first group, the same animals were fear conditioned, exposed to a retrieval cue the next day, and tested for long-term memory 24 h and 1 week after retrieval. In the first group, long-term memory (LTM) was assessed 24 h after retrieval (LTM-1d) and again at 1 week (LTM-7d). In the second group, short-term memory (STM) was assessed 2.5 h after memory retrieval. During each memory test, animals were returned to the retrieval context and exposed to 4 CS presentations, with a pre-CS period of 5 min and an inter-trial interval of 30 s. After testing, animals were returned to the homecage with no further behavioral intervention until the 1 week test.

### Retrieval protocol (with telemetry)

**Fig. 2:**
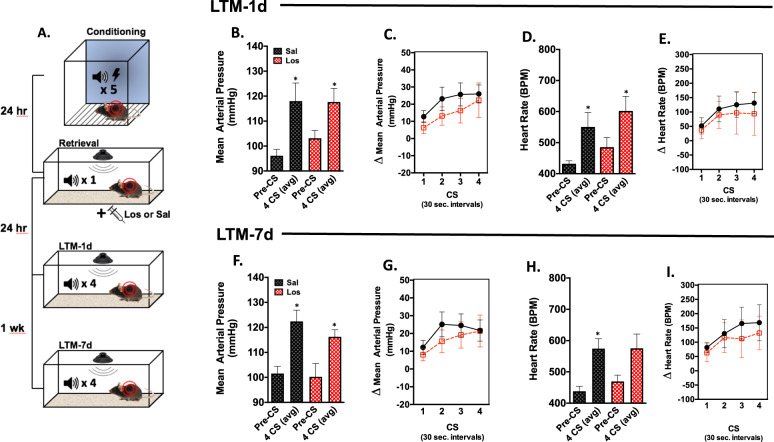
Post-retrieval losartan does not alter recall of a conditioned cardiovascular threat responses. **a** Schematic of the conditioning, retrieval, and testing protocol used in the radio telemetry experiments (A^1^). **b** Average MAP during 5 min baseline (Pre-CS) and throughout 4 CS presentations during the 24 h memory test (LTM-1d). **c** Change in MAP during each 30 s CS relative to the Pre-CS period during the LTM-1d test. **d** Average HR during 5 min baseline (Pre-CS) and throughout 4 CS presentations during the LTM-1d test. **e** Change in HR during each 30 s CS relative to the Pre-CS period during the LTM-1d test. **f** Average MAP during 5 min baseline (Pre-CS) and throughout 4 CS presentations during the 1wk long-term memory (LTM-7d) test. **g** Change in MAP during each 30 s CS relative to the Pre-CS period during the LTM-7d test. **h** Average HR during 5 min baseline (Pre-CS) and throughout 4 CS presentations during the LTM-7d test. **i** Change in HR during each 30 s CS relative to the Pre-CS period during the LTM-7d test. ((*n* = 6 losartan *n* = 4 saline group, *Pre vs. CS, Bonferroni post hoc, *p* < 0.05). (A^1^) Separate groups of mice were utilized for telemetry studies.

Animals equipped with telemeters were fear conditioned as described above. Memory retrieval and subsequent testing for these animals was performed in the homecage to minimize cardiovascular alterations due to handling, and to prevent heightened cardiovascular responses caused by novel context exposure^23^. Twenty-four hours after fear conditioning, the homecage was placed inside a sound attenuating chamber, with a speaker positioned above the bedding. Animals were left undisturbed for approximately 45 min to allow blood pressure and heart rate to return to resting values. The retrieval protocol was remotely initiated and telemetry signals were recorded throughout the session. The behavioral protocol for these animals is depicted in Fig. 2a.

In telemeter-equipped animals, LTM was also assessed using a conditioned cardiovascular threat reactivity test. During this test, the homecage was placed into a sound attenuating chamber and the animals were left undisturbed for 45 min before the testing protocol was initiated. Immediately following each test, the telemetry recording was stopped and the animals were returned to the housing facility. CS-induced changes in blood pressure and heart rate were calculated relative to the pre-CS period. Two weeks after LTM testing, telemeter-equipped animals from both groups were pooled together and injected with either saline or losartan to determine the acute losartan effects on the cardiovascular changes caused by restraint/injection and simulating the post-retrieval losartan injection paradigm (Figs. 1–2). Treatment groups were reversed 3 days later, and the test was repeated. After each injection, the animals were placed back into the home cage where MAP and HR were recorded for 2 h.

### Tissue collection, RNA extraction and whole-genome RNA sequencing

Two separate groups of mice that received either saline or losartan (10 mg/kg) post-retrieval were sacrificed 40 min after the tone with brief exposure to CO_2_ gas. Non-retrieval (NR) control mice did not receive the retrieval cue and were sacrificed at the same timepoint directly from the home cage^23^. Brains were collected, snap frozen and stored at −80 °C^24^. The brains were mounted on Cryostat (CyroStar NX50) and 0.5 mm bilateral tissue punches were taken from the basolateral amygdala (BLA)^25^. For whole genome RNA sequencing, 0.5 mm of bilateral BLA punches were collected and immediately frozen at −80 °C. To extract sufficient amount of RNA from the punches, two mice from either the saline, losartan or non-retrieval groups were pooled together. The resultant 6 mice total per group contributed to the final sample size of *n* = 3 RNA-seq samples/group.

Total RNA was extracted from the pooled BLA samples using Trizol reagent (Sigma) according to the manufacturer’s instructions. RNA quality control (QC), library construction and sequencing were performed by the George Washington University Genomics Core. Samples were constructed into libraries using the Illumina TruSeq Stranded Total RNA Human/Mouse/Rat Library Prep kit (Illumina Inc. San Diego, CA), and were sequenced using an Illumina NextSeq 500 with a High Output Kit v2.5-150 cycles. Genes with a false discovery rate-adjusted Q-score under 0.05 were considered to be statistically significant. Gene ontology annotation was performed using Panther software^26^.

### Quantitative PCR (qPCR)

Gene expression changes were detected from bilateral tissue punches of BLA using relative quantitative-RT-PCR (Applied Biosystems FAST 7500, Foster City, CA). Please refer to the supplement for a detailed description of the methods.

### Data analysis

Prism 6.0 (Graphpad Software Inc., La Jolla, CA, United States) was used for statistical evaluation of behavioral and cardiovascular data. Data were tested for normality using a D’Agnostino–Pearson omnibus normality test, and are presented as the mean ± SEM. One-way ANOVA or *t*-tests were utilized for group comparisons. One-way or two-way analysis of variance (ANOVA) for repeated measures (RM) were employed for comparisons between treatment groups over time. Bonferroni tests were used for post hoc comparisons when appropriate. *P*-values < 0.05 were considered statistically significant.

## Results

### Post-retrieval losartan decreases conditioned auditory fear behavior

To determine whether blockade of AT_1_R during the window of reconsolidation alters the maintenance of conditioned auditory fear memory, mice were fear conditioned and then 24 h later received a single-CS retrieval cue followed by a losartan injection (Fig. 1a). All groups exhibited similar acquisition of fear as measured by percent freezing during the conditioning protocol (Fig. 1b). Prior to drug treatment, animals from both the saline and losartan groups showed a significant increase in freezing during the single CS retrieval cue as indicated by a main effect of Tone (*F*_1, 19_ = 39.09, *p* < 0.0001). There was no main effect of Group (*F*_1, 19_ = 0.03127, *p* = 0.8615) or Group × Tone interaction (*F*_1, 19_ = 2.073, *p* = 0.1662) (Fig. 1c).

STM was assessed 2.5 h after retrieval to determine whether the memory is intact shortly following memory reactivation^13^(Fig. 1d). There was no significant difference in percent freezing between losartan or saline groups (*p* = 0.4178; *n* = 6). The LTM-1d test was then conducted twenty-four hours after retrieval. A significant reduction in freezing behavior was observed in mice that received losartan after memory reactivation (Fig. 1e). Two-way RM ANOVA with the between-subjects factor of Drug (saline and losartan), and the within-subjects factor of Tone (pre-CS and CS) revealed a significant main effect of Tone (*F*_1, 17_ = 87.04, *p* < 0.0001) and a significant Drug × Tone interaction (*F*_1, 17_ = 5.739, *p* = 0.0284) with no main effect of Drug (*F*_1, 17_ = 3.013, *p* = 0.1007). Bonferroni post hoc analysis revealed a significant reduction in freezing in the losartan group (*p* < 0.05) during CS presentation. Memory was assessed one-week later using the same 4 CS cued memory test. At one week, two-way RM ANOVA revealed significant main effects of Tone (*F*_1, 19_ = 59.33, *p* < 0.0001) and Drug (*F*_1, 19_ = 13.33, *p* = 0.0017) with a trend but no significant Drug × Tone interaction (*F*_1, 19_ = 2.282, *p* = 0.1474) (Fig. 1f).

To determine whether the effects of losartan were specific to the process of reconsolidation, we assessed freezing behavior in a separate control cohort of animals that did not undergo retrieval, thus the auditory fear memory was not reactivated (non-retrieval or NR group). Using a similar injection time-course in the absence of a retrieval cue, animals received either saline or losartan and freezing responses were measured 24 h later. Freezing increased in response to the CS as indicated by a significant main effect of Tone (*F*_1, 21_ = 227.9, *p* < 0.0001) (Fig. 1g). Freezing behavior was not significantly different between losartan-treated animals and saline controls (*F*_1, 21_ = 3.823, *p* < 0.0640), and there was no Drug × Tone interaction (*F*_1, 21_ = 0.4378, *p* = 0.5154). Similar results were obtained 1 week later during the LTM-7d test, where there was a main effect of Tone (*F*_1, 18_ = 145.0, *p* < 0.0001), but no significant effect of Drug (*F*_1, 18_ = 1.543, *p* = 0.2301) or Drug × Tone interaction (*F*_1, 18_ = 0.0194, *p* = 0.8908) (Fig. 1h). These results indicate that post-retrieval losartan reduces freezing during both LTM-1d and LTM-7d, and that these effects are dependent upon memory reactivation. Losartan did not affect behavioral responses to the CS at 2.5 h (STM), further suggesting that the observed effects are specific to the neurobiological processes mediating reconsolidation.

### Post-retrieval losartan does not affect conditioned cardiovascular reactivity

To determine whether the reductions in cue-dependent freezing following post-retrieval losartan are accompanied by alterations in the conditioned cardiovascular response, we assessed the cardiovascular state of the animals during threat memory recall. These animals were surgically equipped with telemeters, fear conditioned, and exposed to a single retrieval cue 24 h later as outlined in the schematic (Fig. 2a). Using a conditioned cardiovascular testing paradigm, the animals were exposed to 4 CS presentations while resting in their home-cage 24 h (LTM-1d) and 1 week (LTM-7d) following retrieval. During the LTM-1d test, the auditory cue elicited an increase in MAP (Main effect of Tone *F*_1, 9_ = 20.21, *p* = 0.0015), with no main effect of Drug (*F*_1, 9_ = 0.3128, *p* = 0.5896) and no Drug × Tone interaction (*F*_1, 9_ = 0.8311, *p* = 0.3857) (Fig. 2b, c). A conditioned HR response was also observed during this test, with a significant main effect of Tone (*F*_1, 9_ = 17.19, *p* = 0.0025) but no Drug (*F*_1, 9_ = 1.285, *p* = 0.2862) or Drug × Tone interaction (*F*_1, 9_ = 0.0013, *p* = 0.9724) (Fig. 2d, e).

One week after memory retrieval, the LTM-7d test was performed. MAP increased in both groups during CS presentation, as indicated by a main effect of Tone (*F*_1, 8_ = 18.50, *p* = 0.0026) with no main effect of Drug (*F*_1, 8_ = 0.9117, *p* = 0.3676) and no Drug × Tone interaction (*F*_1,8_ = 0.3162, *p* = 0.5893) (Fig. 2f, g). HR also increased during CS presentation, with a main effect of Tone (*F*_1, 9_ = 13.66, *p* = 0.0050), but no effect of Drug (*F*_1, 9_ = 0.3644, *p* = 0.5610) and no Drug × Tone interaction (*F*_1, 9_ = 0.2175, *p* = 0.6521) (Fig. 2h, i). These findings suggest that conditioned cardiovascular responses in a home-cage environment are unaltered by post-retrieval losartan.

### Losartan attenuates blood pressure increases caused by restraint and injection

**Fig. 3:**
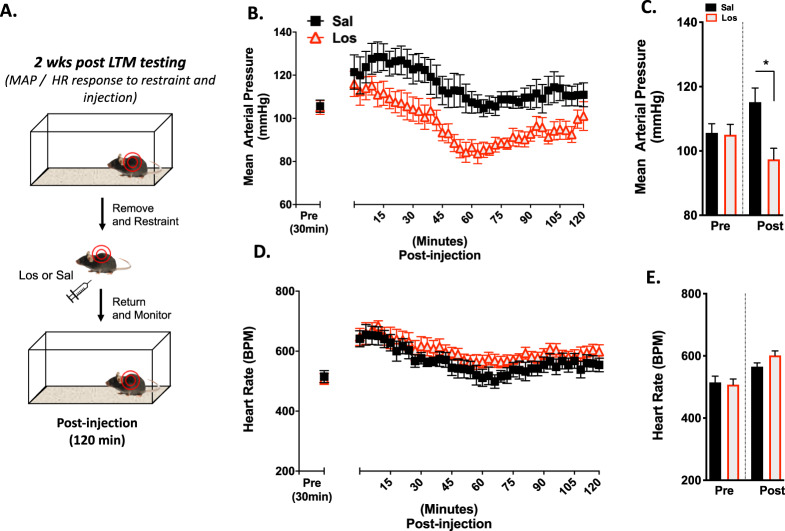
Losartan attenuates increases in blood pressure caused by restraint and injection. **a** Schematic of the handling/restraint and injection protocol used in the telemetry experiments. **b** MAP throughout the 2 h post-injection period (data expressed in 3 min bins). **c** Average mean arterial pressure (MAP) during 30 min pre-injection period and throughout the 2 h post-injection period. **d** HR throughout the 2 h post-injection period (data expressed in 3 min bins). **e** Average heart rate (HR) during 30 min pre-injection period and throughout the 2 h post-injection period (*n* = 12 losartan *n* = 10 saline group, *Pre vs. Post injection, Bonferroni post hoc, *p* < 0.05).

Stress associated with animal handling and restraint during an injection procedure can elicit a robust increase in MAP and HR, and can potentially modulate the strength of reconsolidation^27^. Having demonstrated that post-retrieval losartan reduces freezing behavior at two different timepoints, we next assessed whether these effects might be explained by an altered cardiovascular state via AT_1_R antagonism immediately following retrieval. To address this, we performed additional peripheral losartan injections and monitored the effects on MAP and HR in telemeter-equipped mice. Each mouse was injected with either saline or losartan and immediately placed back in its homecage while telemetry data was collected (Fig. 3a).

Throughout the 2 h post-injection period, there was a main effect of Drug (*F*_1, 20_ = 10.41, *p* = 0.0042) and of Time (*F*_39, 780_ = 10.09, *p* < 0.0001) on MAP, with no significant Drug × Time interaction (*F*_39, 780_ = 0.7456, *p* = 0.8726) (Fig. 3b). Two-way RM ANOVA on the mean MAP during the 2 h following injection showed a significant main effect of Drug (*F*_1, 20_ = 5.703, *p* = 0.0269), and a significant Drug × Time interaction (*F*_1, 20_ = 7.359, *p* = 0.0134), with no main effect of Time (*F*_1, 20_ = 0.08710, *p* = 0.7709) (Fig. 3c). There was a main effect of Time (*F*_39, 780_ = 5.584, *p* < 0.0001) on HR throughout the 2-h post-injection period, with no Drug (*F*_1, 20_ = 3.557, *p* = 0.0739) or Drug × Time interaction (*F*_39, 780_ = 0.2498, *p* > 0.999) (Fig. 3d). Similarly, mean HR following the injection showed a significant main effect of Time (*F*_1, 20_ = 22.46, *p* = 0.0001), but no Drug (*F*_1, 20_ = 0.6690, *p* = 0.4231) or Drug × Time interaction (*F*_1, 20_ = 2.088, *p* = 0.1639) (Fig. 3e). These results demonstrate that acute losartan administration attenuates transient stress-induced (handling and injection) increases in MAP, but not HR. To further interrogate these behavioral and cardiovascular effects, we next sought to determine whether losartan modifies genes within the central nervous system (CNS) that impact fear memory.

### Transcriptomic and protein analysis of the basolateral amygdala (BLA) following post-retrieval losartan

**Fig. 4:**
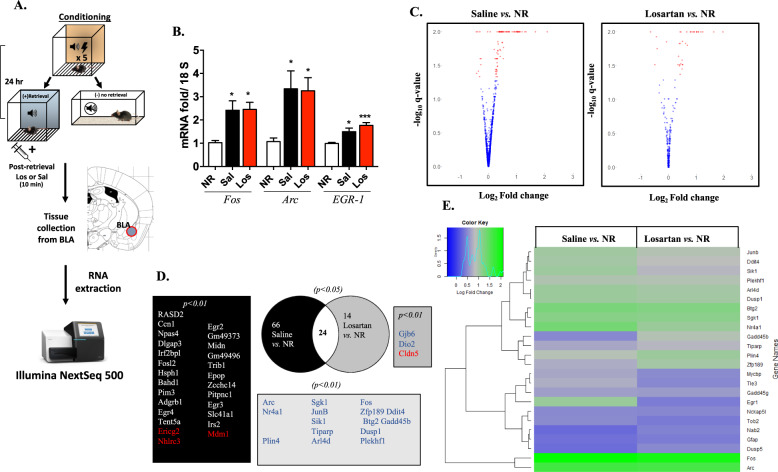
Differential BLA gene expression analysis following post-retrieval losartan. **a** Schematic of the conditioning, retrieval and tissue collection (RNA extraction and sequencing) protocol. **b** Quantitative RT-PCR showing the increased level of immediate early genes (IEG) at 40 min in both saline and losartan groups as compared to NR. (*n* = 8 to 10; Error bars are ±SEM. *p < 0.05, ***p < 0.001 by One-way ANOVA–Tukey’s test). **c** Volcano plots depicting the changes in the expression of 90 genes in saline vs. NR and 38 genes in Losartan vs. NR. Each dot represents a gene with red dots representing genes with corrected *p*-value <0.05 and blue dots with *p*-value >0.05. **d** Venn Diagram illustrates the number and names of the common and uncommon genes between the two comparisons. The genes with significantly altered expression in both saline vs. NR and losartan vs. NR are in white background. Genes with differential expression in only saline vs. NR but not in losartan vs. NR are in black background. Genes with differential expression in only losartan vs. NR but not in saline vs. NR gray background (*p* < 0.05). The names of genes with *p* < 0.01 with significant change in expression are listed for each group in blue or red showing increased and decreased fold change value, respectively. **e** Heat map representing differentially expressed genes in both saline and losartan treated groups after memory retrieval compared to NR. Each line represents a DEG and purple and green indicate the low and high levels of expression, respectively.

The BLA is a key limbic structure for the acquisition, persistence, and loss of fear memory^28,29^. To determine if losartan directly affects immediate early genes (IEGs) expression, we first performed RT-qPCR on BLA tissue, for Fos, Arc and Egr-1, which are essential for the consolidation of fear memories^30^. Using both RT-qPCR and RNA-sequencing, we examined the effects of peripheral losartan on gene transcript changes in the BLA during reconsolidation. We first performed RT-qPCR on BLA tissue to identify activation of immediate early-genes (IEGs): *Fos*, *Arc*, and *Egr-1*, which are essential for the consolidation of fear memories^30^. Irrespective of drug treatment, both retrieval groups showed increased expression (2–3 fold-change) compared to the non-retrieval (NR or “control”) group (Fig. 4b). At the protein level, we also examined ERK1/2 phosphorylation, a key cellular and molecular marker implicated in memory, particularly reconsolidation^31^. Western blot analysis demonstrated that retrieval cue in saline group showed increased pERK1/2 levels in BLA as compared to NR and losartan treatment reduced this pERK1/2 expression to the NR level (Supplemental Fig. 1). We then expanded this analysis using a whole transcriptome RNA-sequencing approach.

For the RNA-sequencing experiment and in a separate cohort of mice, the fear conditioning and memory retrieval protocol were conducted as outlined in schematic (Fig. 4a). Forty minutes following the retrieval cue, all mice were sacrificed and BLA tissue was collected. All the groups of mice had similar fear acquisition and memory retrieval effect (Supplemental Fig. 2). Ninety (90) differentially expressed genes (DEGs) for saline vs. NR and 38 DEGs for losartan vs. NR groups were identified using a *p* < 0.05 statistical cut-off point threshold (Supplementary Table S1, 2). The majority of DEGs (*p* < 0.05; in red) were significantly upregulated in the saline and losartan-treated mice, relative to the control NR group (Fig. 4c). Among the DEGs, 24 common genes (white background) were identified between the 2 comparisons. In saline vs. NR, 66 genes (black background, *p* < 0.05) were uniquely identified as compared to the 14 genes in the losartan vs. NR group (gray background, *p* < 0.05) (Fig. 4d). Heat map analysis also revealed differences in log-fold change between groups (Fig. 4e). In comparing the control vs. saline or losartan groups, there was a large cluster of upregulated genes following cued retrieval and fewer upregulated genes in the losartan group (Fig. 4c–e). Many of these upregulated genes have been previously identified in amygdala-dependent transcriptional regulation of reconsolidation.

**Fig. 5:**
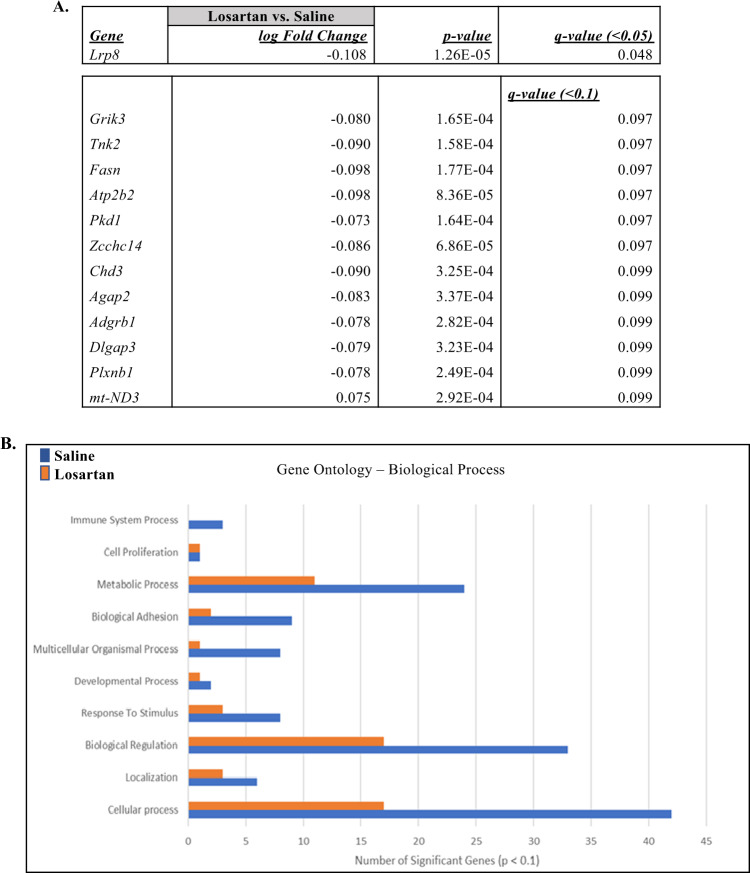
Post-retrieval losartan to saline transcriptomic analysis. **a** Genes differentially expressed in losartan compared to saline group in BLA (*p* < 0.1). **b** Gene Ontology analysis by Panther software for biological processes in between saline and losartan groups (*p* < 0.1).

In additional direct comparative analyses, between saline and losartan groups, we further identified 13 unique DEGs at a statistical threshold of *p* < 0.1 and the gene, *Lrp8* (low density lipoprotein receptor related protein 8) at a statistical threshold *p* < 0.05 (Fig. 5a). This RNA-sequencing result was also confirmed by RT-qPCR where Lrp8 mRNA level was reduced in losartan group compared to saline (*n* = 6, *p* < 0.05) and this effect was specific to BLA region (Supplementary Fig. 3). Interestingly, *Lrp8*, which has been previously implicated in synaptic plasticity and long-term memory formation^32^. Finally, gene ontology analysis on the DEGs, between saline and losartan, showed redistribution of biological processes, mainly biological adhesion, biological regulation and immune system process (Fig. 5b).

## Discussion

Our findings demonstrate that losartan administration shortly after memory retrieval attenuates freezing behavior and modifies differentially expressed genes in the basolateral amygdala, while leaving conditioned cardiovascular responses intact. These results provide new evidence suggesting that post-retrieval losartan may contribute and play an important regulatory role in memory reconsolidation. Overall, these data further our understanding for the underlying neurobiological mechanisms for the role of the brain RAS in fear-related memory.

In addition to losartan, other cardiovascular drug targets^33^ and behavioral interventions^34,35^ are currently being investigated for their potential to disrupt reconsolidation and reduce the strength of fear memories. For example, the FDA-approved β-adrenergic receptor antagonist propranolol has gained significant attention as a potential pharmacotherapeutic for disrupting reconsolidation in humans with PTSD^36^. However, conflicting findings have been reported in both animal models and human clinical trials^33,37–40^. While the clinical efficacy for propranolol as a treatment for PTSD continues to be debated, renewed interest in memory reconsolidation has led to the study of alternative compounds that may be of translational value^41,42^. Similar to propranolol, losartan is an FDA-approved drug used to treat high blood pressure, and it appears that AT_1_R inhibition with losartan can modify various central memory processes in normotensive subjects^4,8,9^. The ability of the RAS to modulate central memory processes may explain early retrospective clinical observations between RAS inhibition and reduced PTSD symptom severity^1^.

To investigate the memory effects of RAS blockade, we used Pavlovian auditory fear conditioning in which a single CS retrieval was used to initiate memory destabilization in mice. The necessity of AT_1_R for this process was determined by pharmacological inhibition during the window of reconsolidation, which in rodents lasts approximately 4–6 h^13,14^. Given the evidence for its role in the formation^18,43,44^, expression^6^, and reconsolidation^16^ of fear memories, we hypothesized that blockade of AT_1_R signaling during the window of reconsolidation would decrease fear responses. In support of this hypothesis, peripheral injection of losartan shortly after memory retrieval reduced freezing behavior in LTM-1d and LTM-7d tests but not during the STM test (2.5 h). Importantly, this reduction in freezing was only observed when losartan was paired with the CS retrieval as losartan had no effect on non-retrieval control animals. These data suggest a reconsolidation-specific drug effect, consistent with previous reports identifying a role for Ang II in memory reconsolidation across species^16,17^.

We recently showed that the acute conditioned blood pressure response to an auditory threat stimulus can be attenuated by extinction training, consisting of repeated re-exposure to the stimulus^20^. Here, we sought to determine whether post-retrieval losartan affects conditioned cardiovascular threat responses independent of extinction learning. In contrast to the effects we observed on freezing behavior, post-retrieval losartan did not significantly modify the conditioned cardiovascular physiological response 24 h or 1 week post-retrieval. This finding should be interpreted with two important features of the study design in mind. First, the retrieval cue in the telemetry animals was presented in the home-cage as opposed to in a novel context. The study was designed this way to allow for measurement of the conditioned response during both retrieval and memory testing, and to remain consistent with common reconsolidation protocols in which retrieval and testing occur in the same context^45^. Given that the ability of amnestic agents to disrupt reconsolidation, this might depend on slight variations in the retrieval procedure^46^, it is possible that the home-cage retrieval cue did not fully engage the process of reconsolidation. The reactivation of a memory is necessary, but not always sufficient to destabilize a memory^47^. Second, it is possible that the conditioned freezing responses need to meet a minimum threshold of reduction before changes in the cardiovascular response are observed. It may be necessary to reduce the fear response by a larger degree before it is detectable in the blood pressure or heart rate components of the conditioned cardiovascular response while strength or intensity of unconditioned stimulus (footshock) should be considered. In either case, an additional goal of the conditioned cardiovascular experiments was to provide an additional objective physiological marker of conditioned fear expression (e.g., blood pressure and HR).

Our current studies suggest that post-retrieval losartan does not alter the conditioned cardiovascular response, although as described above, the home-cage context retrieval procedure in telemetry animals could impact the subsequent conditioned cardiovascular response. Of note, 2 recent clinical studies demonstrate that acute losartan administration enhances extinction learning independent of blood pressure and heart rate measures^8–10^. To our knowledge, this is the first study to use telemetry in a rodent model of memory reconsolidation interrogating the conditioned cardiovascular response following memory retrieval in a home-cage context. Further studies in humans and rodents are needed to determine whether losartan administration (acute or chronic) effects psychophysiological cardiovascular reactivity during threat recall as has been previously shown with propranolol^48^.

Furthermore, recent studies raise the possibility that the physiological or interoceptive emotional state of an animal during reconsolidation may contribute to memory updating. In rats for example, an aversive cue can become a desirable one if the animals are placed in a different physiological state^49^ when they first re-encounter the cue. In our experimental design, mice received an intraperitoneal injection of either losartan or saline 10 min following retrieval. It is possible that the increased stress associated with restraint and handling during the injection could impact the strength of the memory and the expression of associated fear responses. Indeed, we found that the acute post-retrieval administration of losartan significantly blunts the blood pressure response evoked by the restraint and injection procedure. Given the importance of the physiological state of the animal and the role of emotional valence in memory updating^40^, we speculate that this acute and transient blood pressure lowering effect of post-retrieval losartan may contribute to the reductions in freezing observed during the memory tests and losartan dependent gene transcriptional mRNA changes in the BLA.

To further examine potential central mechanisms for post-retrieval losartan’s cardiovascular and behavioral results, we next examined whole genome transcriptional differences in the basolateral amygdala (BLA), a key structure required for acquisition, extinction, and reconsolidation of learned fear^13,28^. Compared to the no-retrieval (NR) control group, several DEGs were identified 40 min after losartan administration and during the window of reconsolidation (38 vs. 90 DEGs), with a large overlap (24 genes) between the two treatment conditions. This overlap may be attributed to the behavioral intervention or the reconsolidation process. Most of the common IEGs between the saline and losartan groups are involved in memory and learning processes and controlled by CREB-mediated phosphorylation. These data support previous studies demonstrating the role of *Egr-1*^50^, *Fos*^51^, and *Arc*^52^ as essential markers for memory consolidation in the lateral amygdala and hippocampus and validate the behavioral retrieval protocol used here.

Results from the whole-genome RNA sequencing analysis in the saline group, identified an upregulation of 90 DEGs following retrieval in the BLA, while in the losartan-treated group, only 38 DEGs following were identified. This reduction in DEGs in the losartan group may suggest that post-retrieval losartan downregulates a majority of the genes that are activated during retrieval. For example, *Egr2* and *Egr3*, involved in neuronal plasticity and immune system activation^53,54^, were differentially expressed only in the saline vs. NR control group comparison, while the losartan group comparison showed differential expression for junction proteins Claudin 5 *(Cldn5)* and Gap junction beta-6 protein (*Gjb*6). Although speculative, this may suggest a role for peripheral losartan in modifying genes important in vascular blood brain barrier permeability, in turn impacting reconsolidation of fear memory, through an unknown mechanism.

In our direct comparison analysis between saline and losartan, there were 13 unique DEGs (*p* < 0.1*)* and one gene, *Lrp8*, which was downregulated at a statistical threshold of *p* < 0.05. Interestingly, Boscarino et al.^55^ recently reported increased expression levels of *Lrp8* in soldiers reporting symptoms of PTSD suggesting that the *Lrp8* Reelin pathway maybe an important blood-based expression profile unique to PTSD diagnosis. Supporting this, previous pre-clinical models have also identified a role for *Lrp8* signaling in long-term memory, cognition and amygdala dependent plasticity via WNT/B-cat signaling^56,57^ Whether or not the effects of losartan on reconsolidation observed in this study are due to changes in BLA-dependent regulation of the *Lrp8* Reelin pathway are unknown and require future study. In both our q-PCR and RNA-sequencing experiments, losartan did not affect immediate early genes (IEGs) (i.e., *Zif268/EGR-1*) which have been previously recognized to be important in memory reconsolidation. Overall, this suggests that post-retrieval losartan has behavioral effects without reducing the transcription and translation of these IEGs (Zif268/EGR-1) and therefore, maybe acting through another downstream molecular pathway (i.e., Lrp8/Wnt; ERK1/2) and mechanism that contributes to regulating reconsolidation or facilitating extinction^4,8^.

We also cannot rule out other potential losartan-dependent neurobiological mechanisms, such as increased conversion and accumulation of various physiologically active brain angiotensin peptides (i.e., AngIV, Ang1-7)^11^, as well as impact on neurotransmitter release (i.e., acetycholine, dopamine, g-amino butyric acid (GABA) and/or neurotrophic factors such as BDNF; all of which indeed could contribute to the underlying mechanisms observed here. Additional studies probing for greater mechanistic understanding are needed. In previous rodent studies, both i.p. and centrally-administered losartan have been shown to have anxiolytic properties, which could impact physiological responses to threat and fear recall and memory^12^. This study used a single dose similar to our previous study^4^ showing no effect on baseline anxiety, which is consistent with other rodent studies^58,59^. Given the 2 hour half-life of the drug and taking into account that our long-term memory tests were conducted 24 h and 1 week following a single injection, it is unlikely that an acute anxiolytic affect is contributing to the behavioral results observed here (Fig. 2). Interestingly, Zhou et al., recently reported in humans that a single acute subcutaneous injection of 50 mg losartan improved early extinction learning and increased ventromedial PFC (vmPFC) activity and functional connectivity between the vmPFC and the basolateral amygdala. These affects were observed beyond the half-life of the drug, thus further supporting the impact of losartan on longer lasting fear memory-dependent mechanisms^8^.

In summary, post-retrieval losartan contributes to long-term reductions in freezing behavior and alterations in central gene expression that occur independent of conditioned cardiovascular reactivity. We also highlight the importance of the physiological state of the animal during the acute window of reconsolidation and the non-specific impact of acute blood pressure lowering due to losartan, during this period. Collectively these studies add to the growing body of evidence describing RAS contributions to fear learning and memory and suggest that targeting the AT_1_R during the reconsolidation phase may be a novel therapeutic approach for fear-related disorders. The ability of systemic AT_1_R inhibition to facilitate both extinction^4,8^, and to modify reconsolidation as shown here, suggest that AT_1_R blockade may be unique in its directional effects on fear memory and support a role for this receptor as a potential pharmacotherapeutic target for maladaptive fear disorders such as PTSD.

## Supplementary information

supplemental figure legends

Supplemental Figure 1

Supplemental Figure 2

Supplemental Figure 3

Supplemental Table 1

Supplemental Table 2
