# Supplementary material for: Evaluation of an angiotensin Type 1 receptor blocker on the reconsolidation of fear memory
Source: Transl Psychiatry. 2020 Oct 27;10:363. doi: 10.1038/s41398-020-01043-6 (PMC7591922; doi:10.1038/s41398-020-01043-6)
Supplement: Supplementary file 1 — supplemental figure legends [file 41398_2020_1043_MOESM1_ESM.docx]

**Supplementary Fig 1:** Freezing behavior data for RNA sequence experiment. (A) Average freezing during the 5^th^ CS presentation of fear conditioning. (B) Freezing behavior before and during the 1CS retrieval cue. Non-retrieval groups (-) did not receive cue exposure (n = 6 per group).

**Supplementary Fig 2:** Effect of post-retrieval losartan on phosphorylation of ERK1/2 in BLA. Representative Western Blot and densitometric analysis of pERK1/2 protein level as compared to total ERK1/2 in BLA for NR, saline and losartan groups 40 minutes after the retrieval cue. Mean optical density, represented as percentage to control NR group (±SEM), shows that losartan treatment significantly inhibits the elevated pERK1/2 expression observed in retrieval saline group almost back to NR level (n=4, *p <0.05 by One-way ANOVA – Tukey’s test) .

**Supplementary Fig 3:** Differential expression of *Lrp8* gene in different brain regions. (A) Quantitative RT-PCR analysis showing decreased levels of *Lrp8* mRNA in losartan treatment group as compared to saline after retrieval at 40 minutes in BLA (n=6, Error bars are ± SEM. *p = 0.0271 by unpaired t -test). (B) Expression of *Lrp8* is significantly increased in losartan group as compared to saline in CA1 hippocampal region (n=6, *p = 0.0364 by unpaired t –test) whereas PVN and PFC do not show any significant difference between groups. (Error bars are ± SEM)

**Supplementary Table 1:** Genes differentially expressed in Saline *vs* NR group in BLA (p<0.05)

**Supplementary Table 2:** Genes differentially expressed in Losartan *vs* NR group in BLA (p<0.05)
