# Supplementary material for: Evaluation of an angiotensin Type 1 receptor blocker on the reconsolidation of fear memory
Source: Transl Psychiatry. 2020 Oct 27;10:363. doi: 10.1038/s41398-020-01043-6 (PMC7591922; doi:10.1038/s41398-020-01043-6)
Supplement: Supplementary file 2 — Supplemental Figure 1 [file 41398_2020_1043_MOESM2_ESM.pdf]

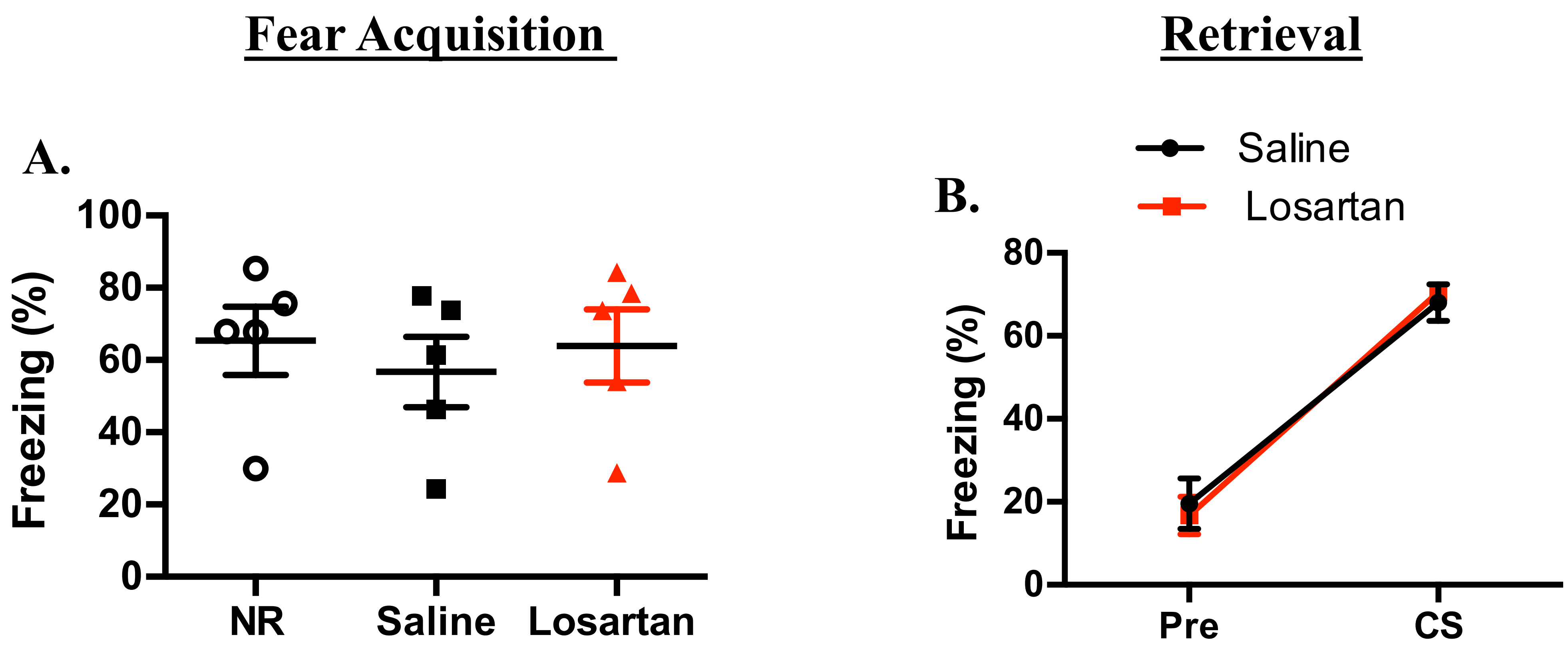

Supplementary Fig 1: Freezing behavior data for RNA sequence experiment. (A) Average freezing during the 5th CS presentation of fear conditioning. (B) Freezing behavior before and during the 1CS retrieval cue. Non-retrieval groups (-) did not receive cue exposure (n = 6 per group).
