# Supplementary material for: Evaluation of an angiotensin Type 1 receptor blocker on the reconsolidation of fear memory
Source: Transl Psychiatry. 2020 Oct 27;10:363. doi: 10.1038/s41398-020-01043-6 (PMC7591922; doi:10.1038/s41398-020-01043-6)
Supplement: Supplementary file 3 — Supplemental Figure 2 [file 41398_2020_1043_MOESM3_ESM.pdf]

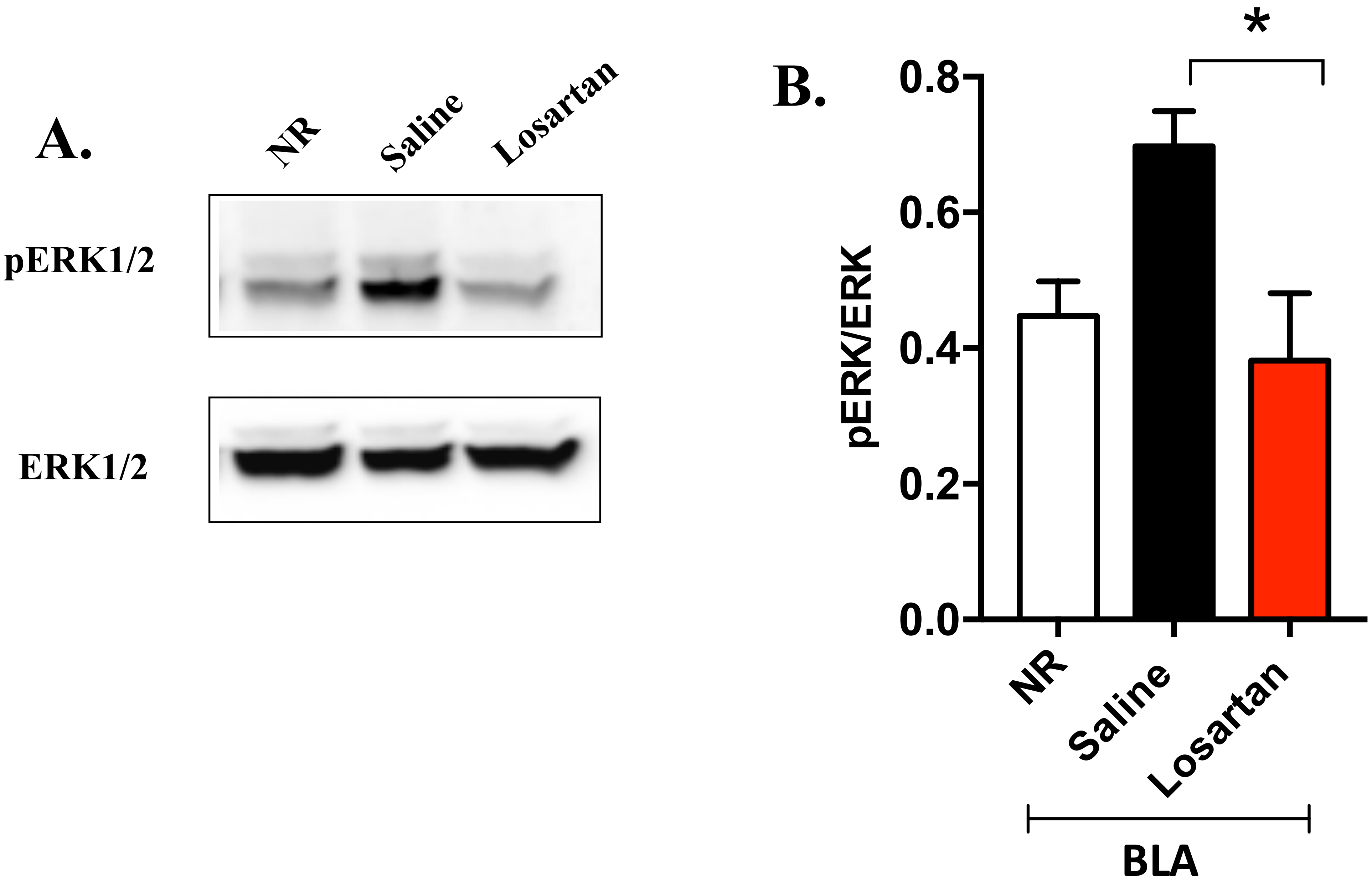

Supplementary Fig 2: Effect of post-retrieval losartan on phosphorylation of ERK1/2 in BLA. Representative Western Blot and densitometric analysis of pERK1/2 protein level as compared to total ERK1/2 in BLA for NR, saline and losartan groups 40 minutes after the retrieval cue. Mean optical density, represented as percentage to control NR group (±SEM), shows that losartan treatment significantly inhibits the elevated pERK1/2 expression observed in retrieval saline group almost back to NR level ($n=4$, $*p < 0.05$ by One-way ANOVA – Tukey's test).
