# Supplementary material for: Evaluation of an angiotensin Type 1 receptor blocker on the reconsolidation of fear memory
Source: Transl Psychiatry. 2020 Oct 27;10:363. doi: 10.1038/s41398-020-01043-6 (PMC7591922; doi:10.1038/s41398-020-01043-6)
Supplement: Supplementary file 4 — Supplemental Figure 3 [file 41398_2020_1043_MOESM4_ESM.pdf]

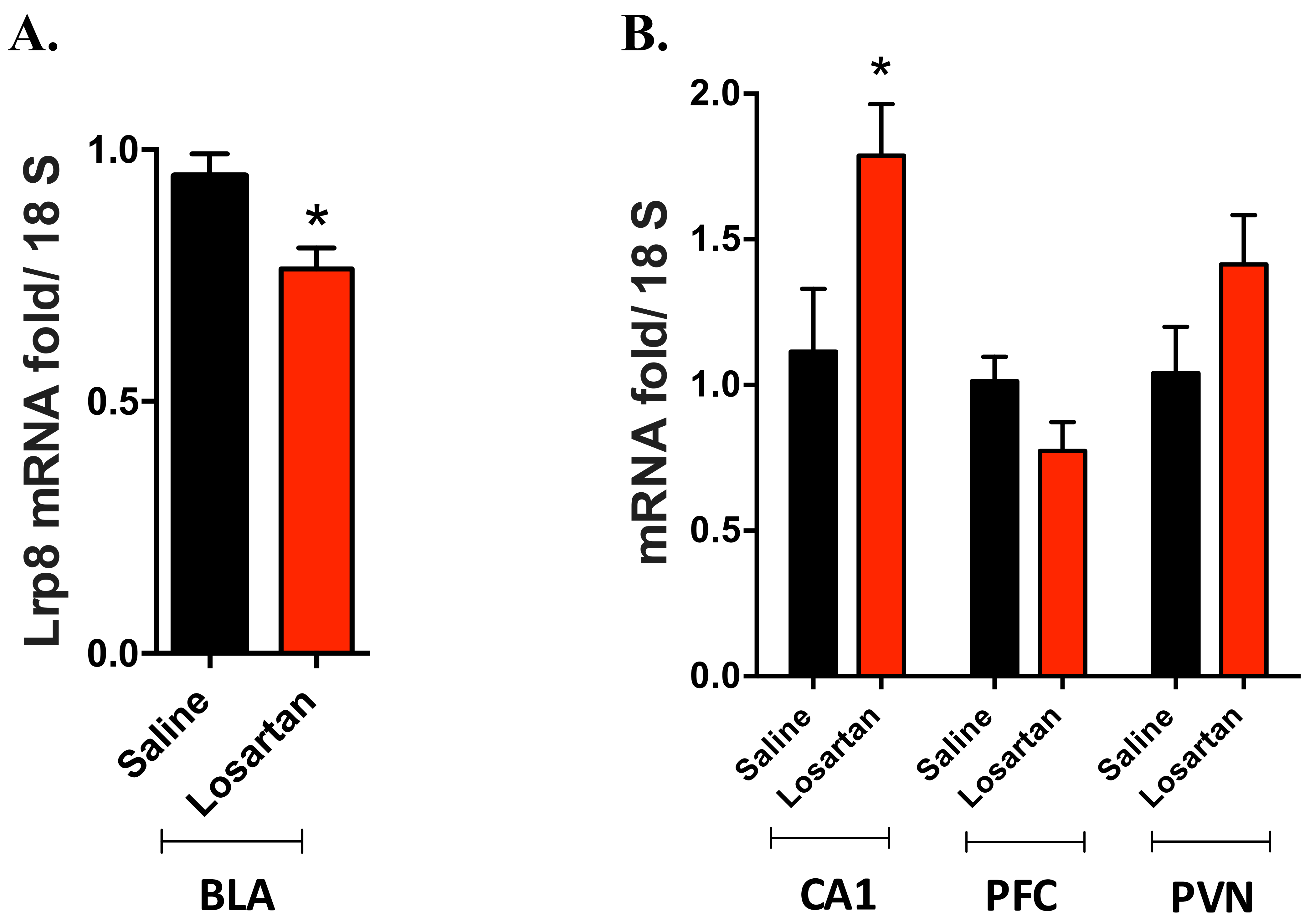

Supplementary Fig 3: Differential expression of *Lrp8* gene in different brain regions. (A) Quantitative RT-PCR analysis showing decreased levels of *Lrp8* mRNA in losartan treatment group as compared to saline after retrieval at 40 minutes in BLA (n=6, Error bars are ± SEM. *p = 0.0271 by unpaired t -test). (B) Expression of *Lrp8* is significantly increased in losartan group as compared to saline in CA1 hippocampal region (n=6, *p = 0.0364 by unpaired t –test) whereas PVN and PFC do not show any significant difference between groups. (Error bars are ± SEM)
