# Supplementary material for: Evaluation of an angiotensin Type 1 receptor blocker on the reconsolidation of fear memory
Source: Transl Psychiatry. 2020 Oct 27;10:363. doi: 10.1038/s41398-020-01043-6 (PMC7591922; doi:10.1038/s41398-020-01043-6)
Supplement: Supplementary file 5 — Supplemental Table 1 [file 41398_2020_1043_MOESM5_ESM.pdf]

	NR *vs.* Saline		
**Gene**	**Log Fold change**	**p-value**	**q-value**
Arc	1.670	4.17E-26	7.47E-22
Fos	2.088	6.52E-24	5.84E-20
Sgk1	1.193	8.56E-23	5.11E-19
Ddit4	1.011	7.09E-19	3.18E-15
Junb	1.031	5.30E-18	1.90E-14
Nr4a1	1.334	8.12E-17	2.42E-13
Sik1	1.050	2.37E-16	6.06E-13
Btg2	1.288	4.89E-13	1.09E-09
Egr1	1.077	6.94E-11	1.38E-07
Dusp1	1.057	1.98E-09	3.55E-06
Zfp189	0.728	1.00E-08	1.64E-05
Tiparp	0.694	2.33E-08	3.47E-05
Rasd2	0.611	2.97E-08	4.09E-05
Ccn1	1.119	1.23E-07	0.0002
Npas4	1.020	1.98E-07	0.0002
Tle3	0.732	2.22E-07	0.0002
Arl4d	1.059	4.30E-07	0.0005
Plin4	0.902	9.13E-07	0.0009
Dlgap3	0.451	8.87E-07	0.0009
Irf2bpl	0.509	1.30E-06	0.0012
Fosl2	0.846	1.60E-06	0.0014
Hsph1	0.383	2.40E-06	0.0019
Ergic2	-0.303	2.32E-06	0.0019
Bahd1	0.478	2.77E-06	0.0020
Tob2	0.507	2.83E-06	0.0020
Fosb	1.002	3.22E-06	0.0021
Pim3	0.444	3.26E-06	0.0021
Plekhf1	0.934	3.07E-06	0.0021
Adgrb1	0.421	3.89E-06	0.0024
Egr4	0.949	4.29E-06	0.0026
Chrm4	0.395	4.45E-06	0.0026
Tent5a	0.542	4.78E-06	0.0027
Mycbp	0.695	4.97E-06	0.0027
Gadd45g	0.638	5.74E-06	0.0030
Egr2	1.111	6.53E-06	0.0032
Gm49373	1.013	7.93E-06	0.0038
Midn	0.405	1.05E-05	0.0048
Rps13-ps2	-0.033	1.07E-05	0.0048
Gm49496	0.062	1.07E-05	0.0048

Trib1	0.479	1.14E-05	0.0049
Arvcf	0.740	5.02E-03	0.0049
Epop	0.556	1.21E-05	0.0050
Nhlrc3	-0.427	1.35E-05	0.0055
Gadd45b	0.488	1.68E-05	0.0067
Zcchc14	0.367	1.76E-05	0.0068
Mycn	0.644	2.04E-05	0.0078
Pitpnc1	0.390	2.38E-05	0.0089
Egr3	0.372	2.57E-05	0.0094
Slc41a1	0.306	2.84E-05	0.0098
Mdm1	-0.384	2.79E-05	0.0098
Irs2	0.463	2.83E-05	0.0098
Gfap	0.333	4.16E-05	0.0140
Nckap5l	0.499	4.37E-05	0.0144
Ier5l	0.489	4.43E-05	0.0144
Actn1	0.347	4.77E-05	0.0152
Rnf165	0.533	5.45E-05	0.0171
Thbs4	0.205	6.04E-05	0.0183
Csrnp1	0.406	5.94E-05	0.0183
Jun	0.430	6.30E-05	0.0188
Tob1	0.334	6.44E-05	0.0189
Rbfox3	0.338	6.63E-05	0.0191
Agap2	0.298	7.19E-05	0.0204
Capn11	0.341	7.62E-05	0.0213
Dact1	0.416	8.45E-05	0.0233
Hlf	0.312	9.91E-05	0.0249
Corin	0.528	0.0001015	0.0249
Sulf2	0.275	0.0001005	0.0249
Dlg4	0.317	9.75E-05	0.0249
Epas1	0.312	9.57E-05	0.0249
Jph3	0.235	0.0001003	0.0249
Nab2	0.294	9.93E-05	0.0249
Rbm39	-0.262	0.00010	0.0249
Zfp945	-0.292	0.00010	0.0249
Utp14b	0.308	0.00012	0.0287
Kctd18	-0.268	0.00013	0.0308
Mn1	0.318	0.00014	0.0324
Hipk2	0.311	0.00014	0.0326
Cacnb3	0.265	0.00016	0.0362
Samd14	0.292	0.00016	0.0362
Mknk2	0.274	0.00016	0.0364
Trp53i11	0.308	0.00017	0.0380
Col5a2	-0.355	0.00018	0.0384

Cnih2	0.251	0.00018	0.0387
Ccdc117	0.291	0.00019	0.0393
Gper1	-0.413	0.00020	0.0412
Tnk2	0.257	0.00021	0.0422
Map7d1	0.289	0.00021	0.0422
Zfp867	-0.313	0.00023	0.0462
Dusp5	0.314	0.00023	0.0463
Flrt1	0.275	0.00024	0.0464

Supplementary Table 1: Differentially expressed BLA genes in saline *vs* NR control group ($p<0.05$)
