# Supplementary material for: Evaluation of an angiotensin Type 1 receptor blocker on the reconsolidation of fear memory
Source: Transl Psychiatry. 2020 Oct 27;10:363. doi: 10.1038/s41398-020-01043-6 (PMC7591922; doi:10.1038/s41398-020-01043-6)
Supplement: Supplementary file 6 — Supplemental Table 2 [file 41398_2020_1043_MOESM6_ESM.pdf]

	**NR *vs.* Losartan**		
**Gene**	**Log Fold change**	**p-value**	**q-value**
Arc	1.635	2.84E-25	4.98E-21
Sgk1	1.170	2.18E-22	1.90E-18
Fos	1.961	2.96E-21	1.72E-17
Junb	0.913	9.03E-15	3.95E-11
Zfp189	1.009	2.14E-14	7.48E-11
Ddit4	0.852	4.33E-14	1.26E-10
Btg2	1.231	2.00E-12	5.01E-09
Nr4a1	1.077	6.75E-12	1.48E-08
Sik1	0.795	3.54E-10	6.89E-07
Gadd45b	0.921	1.34E-09	2.34E-06
Tiparp	0.791	1.67E-09	2.66E-06
Dusp1	1.018	3.57E-09	5.20E-06
Plin4	1.072	1.35E-08	1.81E-05
Arl4d	1.007	8.28E-07	0.0010
Gjb6	0.658	1.58E-06	0.0018
Plekhf1	0.919	3.94E-06	0.0043
Nab2	0.449	7.70E-06	0.0079
Dio2	0.417	8.22E-06	0.0080
Cldn5	-0.647	9.53E-06	0.0088
Tctn1	-0.437	1.36E-05	0.0119
Dusp6	0.501	1.66E-05	0.0139
Gm3591	-0.073	1.77E-05	0.0141
Gfap	0.418	2.12E-05	0.0150
Gadd45g	0.614	2.14E-05	0.0150
Trp53inp1	0.388	2.08E-05	0.0150
Rasl11a	0.679	2.36E-05	0.0159
Heatr6	-0.409	2.73E-05	0.0177
Tsc22d3	0.359	3.66E-05	0.0229
Tle3	0.488	4.35E-05	0.0263
Sox8	0.448	5.06E-05	0.0295
Hps1	-0.498	5.79E-05	0.0306
Mycbp	0.509	6.07E-05	0.0306
Pcgf3	0.338	6.11E-05	0.0306
Dusp5	0.501	5.73E-05	0.0306
Klf10	0.403	5.78E-05	0.0306
Tob2	0.396	6.52E-05	0.0317
Nckap5l	0.485	7.46E-05	0.0353

Egr1	0.418	9.21E-05	0.0424

Supplementary Table 2: Differentially expressed BLA genes in losartan *vs* NR control group ($p<0.05$)
